# Isovitexin-Mediated Regulation of Microglial Polarization in Lipopolysaccharide-Induced Neuroinflammation via Activation of the CaMKKβ/AMPK-PGC-1α Signaling Axis

**DOI:** 10.3389/fimmu.2019.02650

**Published:** 2019-11-14

**Authors:** Bingrun Liu, Bingxu Huang, Guiqiu Hu, Dewei He, Yuhang Li, Xin Ran, Jian Du, Shoupeng Fu, Dianfeng Liu

**Affiliations:** ^1^College of Animal Science and Veterinary Medicine, Jilin University, Changchun, China; ^2^Laboratory of Biomolecular Research, Division of Biology and Chemistry, Paul Scherrer Institute, Villigen, Switzerland

**Keywords:** isovitexin, microglia polarization, neuroinflammation, microglial markers, M1/M2 microglia

## Abstract

Microglia are the brain's immune cells and play an important role in regulating the microenvironment in the central nervous system. Activated microglia are capable of acquiring the pro-inflammatory (M1) phenotype and anti-inflammatory (M2) phenotype. Overactivation of microglia is neurotoxic and may lead to neuroinflammatory brain disorders. Neuroinflammation in the brain plays a crucial role part in the pathophysiology of many psychiatric and neurological diseases. The inhibition of M1 microglia and promotion of M2 microglia was demonstrated to treat and prevent these diseases through reduced neuroinflammation. Isovitexin (IVX) has anti-inflammatory properties and passes through the blood-brain barrier; however, the molecular mechanism that modulates IVX-mediated microglial polarization remains unclear. In BV-2 cells and mouse primary microglia, IVX suppressed the expression of M1 microglial markers, enhanced the expression of M2 microglial markers, and enhanced the release of interleukin 10 (IL-10). IVX promoted the expression of peroxisome proliferator-activated receptor-γ (PPARγ) and PPARγ coactivator-1α (PGC-1α) in LPS-induced microglial activation. The inhibition of PPARγ and PGC-1α attenuated the regulatory effect of IVX in LPS-induced microglial polarization. IVX increased the expression of p-CaMKKβ, p-AMPK, and PGC-1α in BV-2 cells. Inhibition of CaMKKβ with STO-609 or knockdown of CaMKKβ with CaMKKβ siRNA attenuated IVX-mediated M2 microglial polarization in LPS-treated cells. In LPS-treated mice, the inhibition of CaMKKβ and PGC-1α attenuated the IVX-mediated prevention of sickness behavior and enhanction of IVX-mediated M2 microglial polarization. IVX promoted M2 microglial polarization which exerted anti-inflammatory effects on LPS-induced neuroinflammation via the activation of the CaMKKβ/AMPK-PGC-1α signaling axis.

## Introduction

Microglia are derived from myeloid progenitor cells and are the brain's resident macrophages, preventing neuronal injury from secreted neurotoxic mediators (1, 2). Approximately 12% of adult brain cells are ramified microglia (3). Similar to peripheral macrophages, microglia maintain central nervous system (CNS) homeostasis by modulating their phenotypic expression in response to disturbances in the CNS microenvironment. Neuroinflammation induced by activated-microglia leads to neuronal damage and results in a number of brain disorders, including major depressive disorder (MDD) (4, 5).

To simplify the description of results, microglia are classified into two phenotypes: the M1 microglial phenotype is pro-inflammatory and the M2 microglial phenotype is anti-inflammatory. The M1 phenotype promotes the release of pro-inflammatory cytokines with upregulated expression of surface molecules, including CD86 and, CD16/32 (6). The M2 phenotype promotes the expression of anti-inflammatory cytokines, including arginase-1 (Arg1), CD206, CHI3L1 (Ym1 in rodents), and Interleukin 10 (IL-10) that, may decrease neuroinflammation and neuronal injury (7). M2 microglia promote the release of neurotrophic factors and anti-inflammatory molecules that are beneficial for the survival and plasticity of neurons (8). Neuroinflammation causeded by M1 microglial polarization may lead to depressive disorders in lipopolysaccharide (LPS)-treated mice (9). Resveratrol, Compound 3C, and sodium butyrate attenuated M1 microglial activity and promoted M2 microglial activity and this improved MDDs in the presence of neuroinflammatory injury (10–12). Attenuating M1 responses and promoting M2 responses are therapeutic options for the treatment of brain disorders associated with neuroinflammation (13). Suppressing M1 microglia while enhancing M2 microglia may be a neuroprotective therapeutic strategy to improve outcomes in brain disorders. The intricate molecular frameworks of some natural products are sources of inspiration for drug development, especially when evaluating unknown chemotypes (14, 15). Natural products that regulate microglial polarization are valuable in the development of new drugs for the treatment of brain diseases associated with neuroinflammation.

Peroxisome proliferation-activated receptor gamma (PPARγ) has many functions, including immune response regulation, energy metabolism, and mitochondrial functions and PPARγ agonists may exert neuroprotective effects in brain disorders (16–19). PPARγ coactivator-1α (PGC-1α) is a transcriptional co-activator that regulates mitochondrial function and biogenesis, oxidative stress defense, cellular respiration, and expression of myelin basic protein in oligodendrocytes (20–23). PGC-1α overexpression may inhibit the expression of inflammatory mediators in tumor necrosis factor-α (TNF-α)-induced human aortic endothelial cells and may regulate muscle plasticity by inhibiting inflammation (24, 25). PGC-1α knockout in brain tissues attenuated the inhibition of reactive oxygen species (ROS) resulting in neuronal cell damage (26). Thiazolidinedione use increased PPARγ and PGC-1α expression and produced a neuroprotective effect in a Parkinson's disease model (27, 28). PGC-1α activation regulates expression of genes that stimulate mitochondrial biogenesis and neuroinflammation; therefore, targeting transcription factors of PPARγ and PGC-1α may be beneficial in the treatment of brain diseases.

AMP-activated protein kinase (AMPK) is a serine/threonine-protein kinase that acts as a sensor of cellular energy status and regulates various metabolic pathways, including metabolic and functional changes of neurons in brain diseases (29). AMPK is a major regulator of cellular homeostasis and can be phosphorylated and activated in response to an increase in the intracellular AMP-to-ATP ratio (30). Activated AMPK results in the conservation of intracellular ATP levels via multiple downstream pathways to promote M2 microglial polarization (31). Salidroside and berberine activated the AMPK pathway to reduce neuroinflammation associated with M1 microglial polarization and promote M2 microglial polarization (32, 33). AMPK activation is related to the phosphorylation state at threonine (Thr)-172 on the AMPKα subunit (34). AMPK regulates pathogenesis in neuroinflammatory CNS diseases through the suppression of neuroinflammation and neuronal damage; therefore, AMPK pathway activation is a potential therapeutic target (35–37). Calcium/calmodulin-dependent protein kinase kinase-β (CaMKKβ) is the upstream activator of AMPK and regulates inflammatory response in microglia (38). Betulinic acid inhibits neuroinflammation through M2 microglial polarization via CaMKKβ-dependent AMPK activation and CaMKKβ/AMPK pathway activation may prevent microglia-mediated neuroinflammatory brain diseases (39).

Isovitexin (IVX) is a biologically active flavone C-glycosylated derivative of apigenin found in many vegetables, fruits, and herbal medications that possess anti-inflammatory and antioxidant properties (40). IVX is easy to extract, high in content and stable in performance. In rats, IVX crosses the blood-brain barrier and improves memory and anxiolytic-like behaviors via the modulation of GABAA receptors (41, 42). LPS induces M1 microglial polarization that causes M1 microglia to express pro-inflammatory cytokines. IVX acts as an anti-inflammatory and antioxidant in LPS-induced acute lung injury and inhibits inflammatory response in the LPS-activated RAW 264.7 macrophage cell-line (43). Microglial polarization is determined by the expression of M1 and M2 microglial gene markers (44). The effect of IVX on microglial polarization and microglia-mediated neuroinflammation has not been determined. We explored IVX-mediated regulation of microglial polarization and its underlying mechanism *in vitro* and *in vivo*.

## Materials and Methods

### Cell Cultures

BV-2 cells, a murine microglia cell line, were obtained from the Cell Culture Center of the Chinese Academy of Medical Sciences (Beijing, China). The cells were cultured in Dulbecco's Modified Eagle's Medium (DMEM; Gibco, Grand Island, NY, USA) supplemented with 10% fetal bovine serum (FBS; Gibco, Grand Island, NY, USA) and Penicillin-streptomycin (PS) solutions (Beyotime Inst. Biotech, Beijing, China) in 5% CO_2_ at 37°C. After the cells reached ~80% confluence, they were passaged with 0.05% trypsin (Invitrogen, Carlsbad, CA, USA). Cell medium was replaced with serum-free DMEM 6 h prior to treatment with STO-609 (MedChem Express), T0070907 (MedChem Express), SR-18292 (MedChem Express), IVX (>95.0% purity; Sigma-Aldrich, St. Louis, MO, USA), or LPS (Escherichia coli: serotype O55:B5; Sigma-Aldrich, St. Louis, MO, USA). After pretreatment with STO-609 or SR-18292 for a specific time, BV-2 cells were exposed to various concentrations of IVX (dissolved in 0.1% DMSO) for 1 h and then treated with LPS (100 ng/mL) for a specific time.

Mouse primary cortical microglia cells were obtained and cultured from newborn to 24 h old C57BL/6 mice. Briefly, whole mouse brains were removed and placed into ice-cold D-Hanks solution. The meninges and other non-cortical tissues were removed, and the cerebral cortices were cut into 0.5–1 mm^3^ tissue blocks. The tissues were digested in 0.125% trypsin for 10 min at 37°C, followed by the addition of DMEM supplemented with 10% FBS and DNase I, to stop digestion. The cell suspension was filtered through a 70 and 40 μm mesh. The cells were then transferred to 75 cm^2^ poly-L-lysine (PLL-; Sigma-Aldrich, St. Louis, MO, USA)- coated flasks, and cultured in DMEM supplemented with 10% FBS in 5% CO_2_ at 37°C relative humidity. Half of the culture media was changed every 3 days. After 14 days, primary microglia were harvested by shaking the flask for 3 h at 200 rpm and then seeded onto new plates pre-coated with PLL.

### Quantitative PCR

Total RNA was extracted from BV-2 cells and cerebral cortices of mice brains using Trizol reagent (Sigma-Aldrich, St. Louis, MO, USA) according to the manufacturer's protocols. After evaluation by spectrophotometer, 2 μg of RNA was reverse transcribed into cDNA with the PrimeScript® 1st Strand cDNA Synthesis Kit (Invitrogen, Carlsbad, CA, USA) in accordance with manufacturer's protocols. Quantitative PCR (qPCR) was performed by SYBR Green QuantiTect RT-PCR Kit (Invitrogen, Carlsbad, CA, USA). The two-step amplification protocol consisted of 40 cycles of denaturation at 95°C for 10 s, annealing at 60°C for 30 s, and extension at 72°C for 30 s. The relative levels of gene expression for each mRNA were calculated by normalization to β-actin mRNA expression levels according to the2^−ΔΔCT^ method. The primer sequences designed in Sangon Biotech (Shanghai, China) for the tested genes are shown in Table 1.

**Table 1:** Primers for real-time RT-PCR.

**Gene**	**Sense (5^**′**^-3^**′**^)**	**Anti-Sense (5^**′**^-3^**′**^)**
β-actin	GTCAGGTCATCACTATCGGCAAT	AGAGGTCTTTACGGATGTCAACGT
TNF-α	CCCCAAAGGGATGAGAAGTTC	CCTCCACTTGGTGGTTTGCT
IL-6	CCAGAAACCGCTATGAAGTTCC	GTTGGGAGTGGTATCCTCTGTGA
IL-1β	GTTCCCATTAGACAACTGCACTACAG	GTCGTTGCTTGGTTCTCCTTGTA
iNOS	GAACTGTAGCACAGCACAGGAAAT	CGTACCGGATGAGCTGTGAAT
COX-2	CAGTTTATGTTGTCTGTCCAGAGTTTC	CCAGCACTTCACCCATCAGTT
Arg-1	GTGAAGAACCCACGGTCTGT	GCCAGAGATGCTTCCAACTG
CD206	CTTCGGGCCTTTGGAATAAT	TAGAAGAGCCCTTGGGTTGA
YM1/2	CAGGGTAATGAGTGGGTTGG	CACGGCACCTCCTAAATTGT
PPARγ	ACAGGAAAGACAACGGACAAATCA	CTTCTACGGATCGAAACTGGCAC
PGC-1α	TGATGTGAATGACTTGGATACAGACA	GCTCATTGTTGTACTGGTTGGATATG

### Measurement of IL-10 by ELISA

ELISA was used to detect IL-10 level in the media cultured BV-2 cells and mouse primary microglia according to the manufacturer's protocols (45). Briefly, when the cells reached 80% confluence they were treated with IVX for 2 h followed by treatment with LPS (100 ng/mL) for 24 h. The cell culture media was harvested and then detected by the ELISA kits obtained from BioLegend.

### Fluorescent Immunocytochemistry

The primary microglia were harvested and seeded onto PLL-coated inserts in 24-well plates and cultured 24 h. The purity of primary microglia was detected by IBA-1 (RRID:AB_2636859, 1:200) and GFAP (RRID:AB_296804, 1:500) staining, as previously described. Representative images were shown.

### Knockdown of PPARγ, PGC-1α, and CaMKKβ in Mouse Primary Microglial Cells by siRNA

The primary microglia were cultured in 6-well plates for 12 h and then transfected with scrambled control siRNA, PPARγ siRNA, PGC-1α siRNA, or CaMKKβ siRNA (Genepharma, Shanghai, China). Lipofectamine 3000 (Invitrogen, Camarillo, CA) and siRNA were premixed in Opti-MEM (Invitrogen, Camarillo, CA) according to the manufacturer's instructions and then applied to the cells. After 24 h of transfection, Opti-MEM was replaced by DMEM medium without FBS. Then primary microglial cells were pretreated with IVX, followed by treatment with LPS (100 ng/mL) for another 6 h or 12 h. All siRNA sense strands are listed in Table 2.

**Table 2:** Sequence of target gene siRNA.

**Gene**	**Sense (5^**′**^-3^**′**^)**
Control siRNA	UUCUCCGAACGUGUCACGUTT
PPARγ siRNA	GCGAUCUUGACAGGAAAGATT
PGC-1α siRNA	CCGCAAUUCUCCCUUGUAUTT
CaMKKβ siRNA	CAGGAGAUUGCUAUCCUCAAAT

### Experimental Animals and Protocols

Our research was conducted in accordance with approved animal treatment protocols and guidelines established by the Institutional Animal Care and Use Committee of Jilin University (Changchun, China) (approved on February 27, 2015; Protocol No. 2015047). We have done our best to minimize animal suffering and to reduce the number of animals used. Male C57BL/6 mice (8–10 weeks and 20–25 g) were obtained from Liaoning Changsheng Technology Industrial (Liaoning, China). The mice were housed under environmentally controlled conditions (24 ± 1°C under a 12 h light-dark cycle with a relative humidity of ~50–80%). The mice were supplied with tap water and food. The mice were randomly divided into the following six groups (8–10 mice per group): control (vehicle solution); inhibitor (STO-609/SR-18292, 5 μg dissolved in 2 μL artificial cerebrospinal fluid, ACSF); IVX; LPS; LPS + IVX; inhibitor (STO-609/SR-18292) + IVX + LPS. Mice were anesthetized and positioned in a stereotaxic apparatus and then their lateral ventricles were injected with ACSF containing STO-609, SR-18292, or negative control (2.0 μL, 0.20 μL/min). The stereotactic coordinates from the skull surface were: anterior-posterior (AP) = + 0.5 mm, mediolateral (ML) = – 0.8 mm, and dorsoventral (DV) = – 2.5 mm. IVX (2.5 mg/mL) or saline was injected intraperitoneally (10 mg/kg) into mice once daily for 3 days. After injection with IVX or saline for 2 h on the third day, mice were injected intraperitoneally with LPS (0.33 mg/kg) or saline. After injection with LPS for 24 h, body weight changes in the mice were measured and then open field tests were conducted. After testing, mice were anesthetized with a ketamine/xylazine (150:10 mg/kg) mixture and intracardially perfused with saline. The cerebral cortices were isolated and cryopreserved at −80°C for further studies.

### Western Blotting

Cerebral cortices and microglia cells were lysed in RIPA lysis buffer (Beyotime Inst. Biotech, Beijing, China) containing protease and phosphatase inhibitor (cocktails and phenylmethylsulphonyl fluoride) and western blotting was performed as previously described (30, 31). Briefly, equal amounts of protein (40 μg) were loaded and separated by 12% SDS-PAGE, then transferred to polyvinylidene difluoride (PVDF) membranes. The PVDF membranes were blocked with 5% skim milk-TBST for 2 h at room temperature and then incubated with primary antibodies: Rabbit anti-PPARγ (RRID:AB_10596794, 1:1,000), β-actin (RRID:AB_2289225, 1:3,000), CD206 (RRID:AB_10597232, 1:1,000), Arg-1(RRID:AB_2289842, 1:1,000), Mouse anti-PGC-1α (RRID:AB_2631201, 1:2,000), Rabbit anti-AMPK (RRID:AB_10624867, 1:1,000), p-AMPK (RRID:AB_331250, Thr172) (1:1,000), CaMKKβ (RRID:AB_2798771, 1:1,000), p-CaMKKβ (RRID:AB_2798769, 1:1,000), NF-κB p65 (RRID:AB_10828935, 1:2,000) and p-NF-κB p65 (RRID:AB_331284, 1:1,000), Rabbit anti-Iba-1 (RRID:AB_2636859, 1:1,000), TNF-α (RRID:AB_778525, 1:1,000), IL-10 (RRID:AB_308826, 1:1,000), GFAP (RRID:AB_296804, 1:200), and iNOS (RRID:AB_881438, 1:1,000) antibodies (dissolved in 5% bovine serum albumin-TBST) for 12 h at 4°C; and then incubated with secondary antibodies: goat anti-rabbit (Boster, 1:2,000) and goat anti-mouse (Boster, 1:2,000) (dissolved in 5% skim milk-TBST) for 1 h at 25°C. The blots were incubated and then visualized with ECL Western blot detection reagents (Amersham Pharmacia Biotech). Next, the blots were determined with ECL Western blot detection reagents (Amersham Pharmacia Biotech), and performed in accordance with standard protocols (46). To determine the effect of IVX on the CaMKKβ/AMPK-PGC-1α signaling axis, BV-2 cells were treated with a CaMKKβ inhibitor (STO-609) or an AMPK inhibitor (Compound C; MedChem Express) and analyzed using western blotting as previously described.

### Determination of Sickness Behavior, Bodyweight Change, and Locomotor Activity

In mice, the LPS-induced neuroinflammatory model has been reported and demonstrated (32, 33). Two characteristics of sickness behavior are weight loss and reduced locomotor activity. Mouse bodyweight was measured using a weight scale at the beginning and end of the experiment. Locomotor activity was measured using the open field test. A square (60 × 60 × 35 cm), open field box (Any-maze, Stoelting Co) was performed to inner and outer zones in base. Mice were gently placed into a corner of the open field box for 5 min. During this time the total distance traveled and time spent in the inner zones of the box were recorded with an overhead camera and analyzed using TopScan software (Any-maze, Stoelting Co) according to the manufacturer's instructions.

### Statistical Analysis

Significance testing was conducted using SPSS 19.0 software (IBM). All data represented repeated experiments and are expressed as means ± SEM. Multi-group comparisons were performed using a one-way ANOVA, with the least significant difference (LSD) method. Differences were considered statistically significant at *p* < *0.05* and *p* < *0.01*.

## Results

### IVX Suppressed M1 Microglial Polarization and Promoted M2 Microglial Polarization in LPS-Activated BV-2 Cells and Mouse Primary Microglia

**Figure 1:**
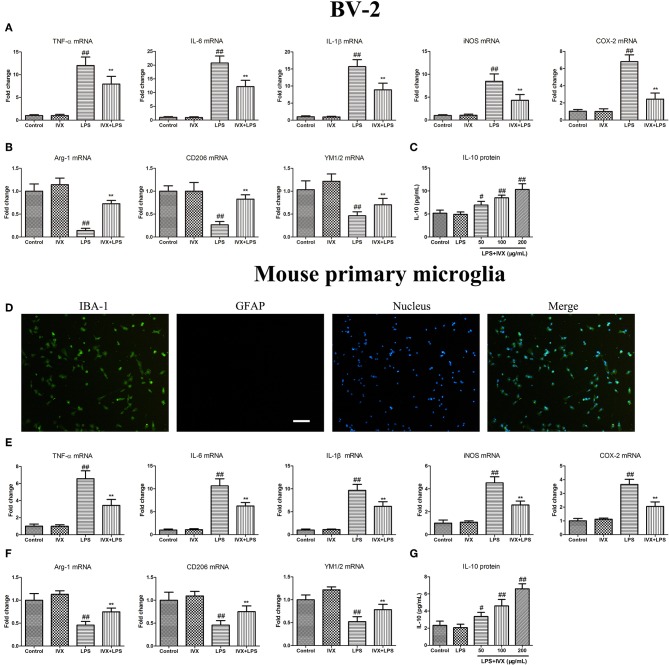
Isovitexin (IVX) inhibited M1 microglial polarization and promoted M2 microglial polarization in LPS-activated BV-2 cells and mouse primary microglia. **(D)** The purity of mouse primary microglia was detected by Fluorescent immunocytochemistry. BV-2 cells and mouse primary microglia were pretreated with IVX (200 μg/mL) for 2 h and then treated with LPS (100 ng/mL) for 6 h and 24 h. After 6 h, cells were then harvested and the mRNA expression of M1 microglial markers (TNF-α, IL-6, IL-1β, iNOS, and COX-2) and M2 microglia markers (Arg-1, CD206, and YM1/2) were detected by RT-PCR. **(A,E)** IVX inhibited the mRNA expression of M1 microglial markers (TNF-α, IL-6, IL-1β, iNOS, and COX-2) in LPS-activated BV-2 cells and mouse primary microglia. **(B,F)** IVX attenuated the decrease of M2 microglial markers (Arg-1, CD206, and YM1/2) in LPS-activated BV-2 cells and mouse primary microglia. After 24 h, cells medium were harvested and the release of IL-10 were investigated by ELISA. **(C,G)** IVX enhanced the release of IL-10 in LPS-activated BV-2 cells and mouse primary microglia. The experiments were conducted in triplicate and repeated at least three times. Values are expressed as mean ± SEM (*n* = 4 in each group). ^#^*p* < *0.05*, ^*##*^*p* < 0.01, vs. control group; **p < 0.01 vs. LPS group.

MTT assays revealed that at concentrations ≤ 200 μg/mL IVX was not cytotoxic to BV-2 cells or mouse primary microglia (no shown). Immunofluorescence analyses revealed the purity of primary microglia was ≥98% (Figure 1D). IVX's ability to suppress LPS-induced M1 microglial polarization in BV-2 cells and mouse primary microglia was tested. LPS treatment increased the mRNA expression of inflammatory cytokines (M1 markers) TNF-α, IL-6, and IL-1β, and of proinflammatory enzymes (M1 markers) iNOS and COX-2 and IVX pretreatment suppressed the mRNA expression of these inflammatory cytokines (Figures 1A,E). LPS-stimulation M2 microglial polarization decreased the mRNA expression of M2 markers (Arg-1, CD206, and YM1/2) in BV-2 cells and mouse primary microglia and IVX pretreatment enhanced the mRNA expression of these markers (Figures 1B,F). ELISA showed that IVX treatment enhanced the release of IL-10 in LPS-treated BV-2 cells and mouse primary microglia (Figures 1C,G). IVX suppressed M1 microglial polarization and promoted M2 microglial polarization, suppressing the inflammatory response in activated microglia.

### IVX Enhanced the Expression of PPARγ and PGC-1α in LPS-Activated BV2 Cells and Mouse Primary Microglia

**Figure 2:**
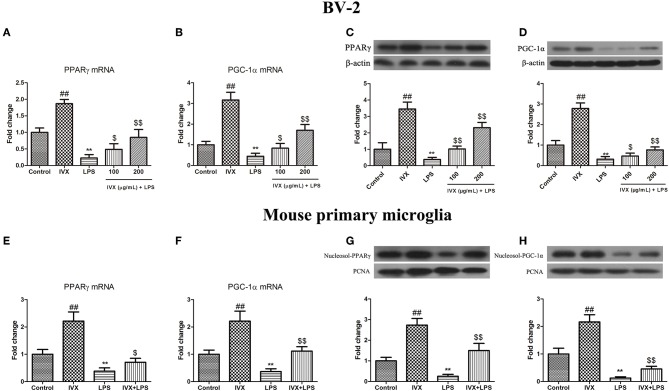
IVX suppressed the mRNA and protein expression of PPARγ and PGC-1α in LPS-activated BV-2 cells and mouse primary microglia. **(A,B,E,F)** RT-PCR revealed that IVX up-regulated the mRNA expression of PPARγ and PGC-1α in LPS-activated BV-2 cells and mouse primary microglia. Cells were pretreated with IVX (200 μg/mL) for 2 h and then stimulated with LPS (100 ng/mL) for 6 h. **(C,D)** Western blotting revealed IVX enhanced the protein expression of PPARγ and PGC-1α in LPS-activated BV-2 cells. BV-2 cells were pretreated with IVX (200 μg/mL) for 2 h and then stimulated with LPS (100 ng/mL) for 12 h. **(G,H)** Western blotting revealed IVX enhanced the nuclear protein expression of PPARγ and PGC-1α in LPS-activated mouse primary microglia. Cells were pretreated with IVX (200 μg/mL) for 2 h and then stimulated with LPS (100 ng/mL) for 12 h. The experiments were conducted in triplicate and repeated at least three times. Values are expressed as mean ± SEM (*n* = 4 in each group). ^*##*^*p* < 0.01, vs. control group; **p < 0.01 vs. control group; ^$^*p* < 0.05 and ^$$^*p* < 0.01 vs. LPS group.

Promotion of PPARγ and PGC-1α suppressed microglial activation and reduced the expression of pro-inflammatory mediators. LPS treatment decreased the gene expression of PPARγ and PGC-1α in BV-2 cells and mouse primary microglia (Figures 2A–D). IVX-mediated microglial polarization in LPS-treated BV-2 cells and mouse primary microglia enhanced the gene expression of PPARγ and PGC-1α (Figures 2A,B,E,F). LPS treatment decreased the protein expression of PPARγ and PGC-1α, while IVX counteracted the effects of LPS on the protein expression of PPARγ and PGC-1α in LPS-treated BV-2 cells (Figures 2C,D). LPS decreased the nuclear protein expression of PPARγ and PGC-1α, while IVX counteracted the effects of LPS on the nuclear protein expression of PPARγ and PGC-1α in LPS-treated mouse primary microglia (Figures 2G,H), suggesting IVX can activate PPARγ and PGC-1α in microglia.

### PPARγ Activation Is Involved in the IVX-Mediated Microglial Polarization of BV2 Cells and Mouse Primary Microglia

**Figure 3:**
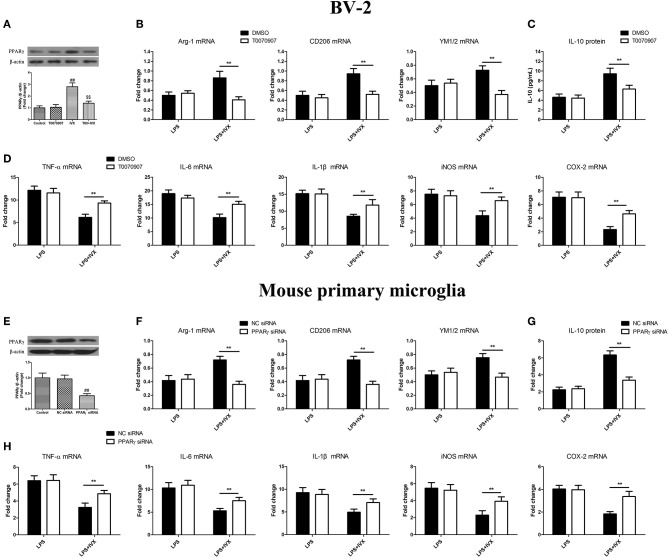
PPARγ activation is involved in IVX-mediated microglial polarization in LPS- induced BV-2 cells and mouse primary microglia. **(A)** After treatment with T0070907 (an inhibitor of PPARγ) for 2 h and PPARγ siRNA for 24 h and then IVX for 2 h in BV-2 cells and mouse primary microglia, the protein expression of PPARγ was measured with Western blotting. Following pretreatment with 5 μM T0070907 for 2 h and PPARγ siRNA for 24 h, cells were treated with IVX (200 μg/mL) for 2 h and then stimulated with LPS (100 ng/mL) for 6 h. **(E)** The protein expression of PPARγ was detected via Western blotting. **(B,F)** The mRNA expression of M2 microglial markers (Arg-1, CD206, and YM1/2) were detected via RT-PCR. **(D,H)** The mRNA expression of M1 microglial markers (TNF-α, IL-6, IL-1β, iNOS, and COX-2) were detected via RT-PCR. Pretreatment with 5 μM T0070907 for 2 h and PPARγ siRNA for 24 h, cells were treated with IVX (200 μg/mL) for 2 h and then stimulated with LPS (100 ng/mL) for 24 h. **(C,G)** The release of IL-10 was measured with ELISA. The experiments were conducted in triplicate and repeated at least three times. Values are expressed as mean ± SEM (*n* = 4 in each group). ^*##*^*p* < 0.01, vs. control group; ^$$^p<0.01, vs. IVX group; **p< 0.01, T0070907 or PPARγ siRNA + LPS + IVX group vs. LPS + IVX group.

PPARγ inhibitor T0070907 was used to block PPARγ activity (Figure 3A). PPARγ protein expression was decreased in mouse primary microglia when transfected with PPARγ siRNA for 24 h (Figure 3E). In LPS-induced BV-2 cells and mouse primary microglia, 5 μM T0070907 (PPARγ inhibitor) and PPARγ siRNA did not affect IVX-mediated microglial polarization as measured by the expression of M1 (TNF-α, IL-6, IL-1β, iNOS, and COX-2 mRNA) and M2 (Arg-1, CD206, and YM1/2 mRNA) markers (Figures 3B,D,F,H). In LPS-induced polarized BV-2 cells and mouse primary microglia, pretreatment with T0070907 and PPARγ siRNA attenuated the inhibition of M1 markers (TNF-α, IL-6, IL-1β, iNOS, and COX-2 mRNA) by IVX and T0070907 and PPARγ siRNA treatment attenuated the enhancement of M2 markers (Arg-1, CD206, and YM1/2 mRNA) by IVX (Figures 3B,D,F,H), revealing that IVX-mediated PPARγ expression regulates microglial polarization. In LPS-induced polarized BV-2 cells and mouse primary microglia, pretreatment with T0070907 and PPARγ siRNA attenuated the release of IL-10 by IVX (Figures 3C,G), suggesting that IVX enhances M2 microglial polarization via the activation of PPARγ.

### PGC-1α Activation Is Involved in the IVX-Mediated Microglial Polarization of BV2 Cells and Mouse Primary Microglia

**Figure 4:**
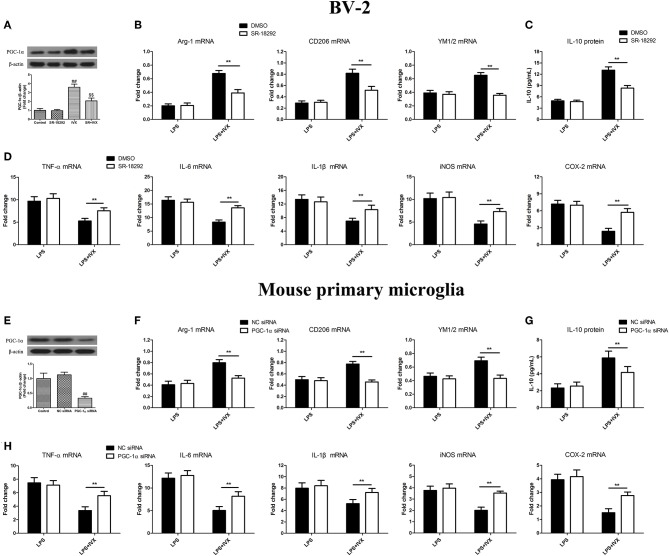
PGC-1α activation is involved in IVX-mediated microglial polarization in LPS- induced BV-2 cells and mouse primary microglia. **(A)** After treatment with SR-18292 (an inhibitor of PGC-1α) for 3 h and PGC-1α siRNA for 24 h and then IVX for 2 h in BV-2 cells and mouse primary microglia, the protein expression of PGC-1α was measured with Western blotting. Following pretreatment with 2.5 μM SR-18292 for 3 h and PGC-1α siRNA for 24 h, cells were treated with IVX (200 μg/mL) for 2 h and then stimulated with LPS (100 ng/mL) for 6 h. **(E)** The protein expression of PGC-1α was detected via Western blotting. **(B,F)** The mRNA expression of M2 microglial markers (Arg-1, CD206, and YM1/2) were detected via RT-PCR. **(D,H)** The mRNA expression of M1 microglial markers (TNF-α, IL-6, IL-1β, iNOS, and COX-2) were detected via RT-PCR. Pretreatment with 2.5 μM SR-18292 for 3 h and PGC-1α siRNA for 24 h, cells were treated with IVX (200 μg/mL) for 2 h and then stimulated with LPS (100 ng/mL) for 24 h. **(C,G)** The release of IL-10 was measured with ELISA. The experiments were conducted in triplicate and repeated at least three times. Values are expressed as mean ± SEM (*n* = 4 in each group). ^*##*^*p* < 0.01, vs. control group; ^$$^*p* < 0.01, vs. IVX group; **p < 0.01, SR-18292 or PGC-1α siRNA + LPS + IVX group vs. LPS + IVX group.

PGC-1α inhibitor SR-18292 was used to block PGC-1α activity (Figure 4A). PGC-1α protein expression was decreased in mouse primary microglia when transfected with PGC-1α siRNA for 24 h (Figure 4E). In LPS-induced BV-2 cells and mouse primary microglia, 2.5 μM SR-18292 (PGC-1α inhibitor) and PGC-1α siRNA did not affect IVX-mediated microglial polarization as measured by the expression of M1 (TNF-α, IL-6, IL-1β, iNOS, and COX-2 mRNA) and M2 (Arg-1, CD206, and YM1/2 mRNA) markers (Figures 4B,D,F,H). In LPS-induced polarized BV-2 cells and mouse primary microglia, pretreatment with SR-18292 and PGC-1α siRNA attenuated the inhibition of M1 markers (TNF-α, IL-6, IL-1β, iNOS, and COX-2 mRNA) by IVX and SR-18292 and PGC-1α siRNA treatment attenuated the enhancement of M2 markers (Arg-1, CD206, and YM1/2 mRNA) by IVX (Figures 4B,D,F,H), revealing that IVX-mediated PGC-1α expression regulates microglial polarization. In LPS-induced polarized BV-2 cells and mouse primary microglia, pretreatment with SR-18292 and PGC-1α siRNA attenuated the release of IL-10 by IVX (Figures 4C,G), suggesting that IVX enhances M2 microglial polarization via the activation of PGC-1α.

### IVX Regulated the Expression of PGC-1α in BV2 Cells via CaMKKβ-Dependent AMPK Activation

**Figure 5:**
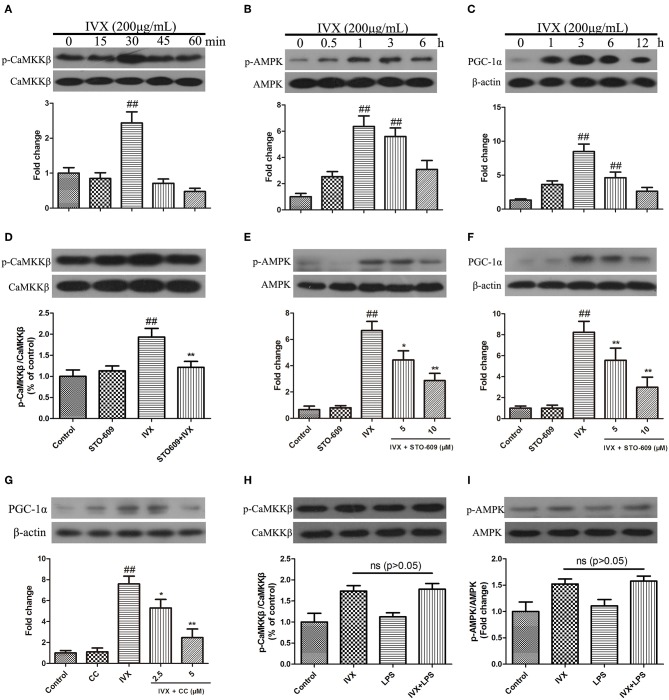
IVX enhanced the expression of PGC-1α via the CaMKKβ-dependent AMPK signaling pathway in BV-2 cells. **(A–C)** BV-2 cells were treated with IVX (200 μg/mL) for different time. Cells were then harvested and the protein expression of p-CaMKKβ **(A)**, p-AMPK **(B)**, and PGC-1α **(C)** was detected by western blotting. **(D)** BV-2 cells were treated with 10 μM of STO-609 (a CaMKKβ inhibitor) for 2 h and then treated with IVX for 30 min, the protein expression of p-CaMKKβ was detected by Western blotting. **(E)** Cells were pretreated with STO-609 (5 and 10 μM) for 2 h, followed by stimulation with IVX (200 μg/mL) for 1 h. The protein expression of p-AMPK and AMPK were detected by western blotting. **(F)** BV-2 cells were pretreated with the STO-609 (5 and 10 μM) for 2 h, followed by stimulation with IVX (200 μg/mL) for 3 h. The protein expression of PGC-1α was detected by western blotting. **(G)** Cells were pretreated with the 2.5 and 5 μM Compound C (CC, an AMPK inhibitor) for 2 h, and then treated with IVX (200 μg/mL) for 3 h. The protein expression of PGC-1α was determined by western blotting. **(H)** After treatment with IVX (200 μg/mL) and IVX (200 μg/mL) + LPS (100 ng/mL) for 30 min, the protein expression of p-CaMKKβ was investigated. **(I)** After treatment with IVX (200 μg/mL) and IVX (200 μg/mL) + LPS (100 ng/mL) for 3 h, the protein expression of p-AMPK was investigated. β-actin was utilized as an internal control and the experiments were conducted in triplicate and repeated at least three times. Values are expressed as mean ± SEM (*n* = 4 in each group). ^*##*^*p* < 0.01, vs. control group; *p < 0.05 and **p < 0.01 vs. IVX group.

In BV-2 cells, IVX (200 μg/mL) enhanced the phosphorylation of CaMKKβ for 30 min, increased p-AMPK expression for 1 h, and increased PGC-1α expression for 3 h (Figures 5A–C). CaMKKβ inhibitor STO-609 was used to block p-CaMKKβ activity (Figure 5D). Western blotting revealed that 10 μM STO-609 did not affect the expression of p-AMPK and PGC-1α in BV-2 cells and pretreatment with STO-609 (5 or 10 μM) attenuated IVX-induced p-AMPK and PGC-1α expression in BV-2 cells (Figures 5E,F). Pretreatment with compound C (2.5 or 5 μM) attenuated IVX-induced PGC-1α expression in BV-2 cells (Figure 5G). LPS treatment has no effct on the phosphorylation of CaMKKβ and AMPK in BV-2 cells (Figures 5H,I), indicating that IVX independently activates the CaMKKβ/AMPK signaling pathway in LPS-induced BV-2 cells.

### CaMKKβ Activation Is Involved in IVX-Mediated Microglial Polarization of BV2 Cells and Mouse Primary Microglia

**Figure 6:**
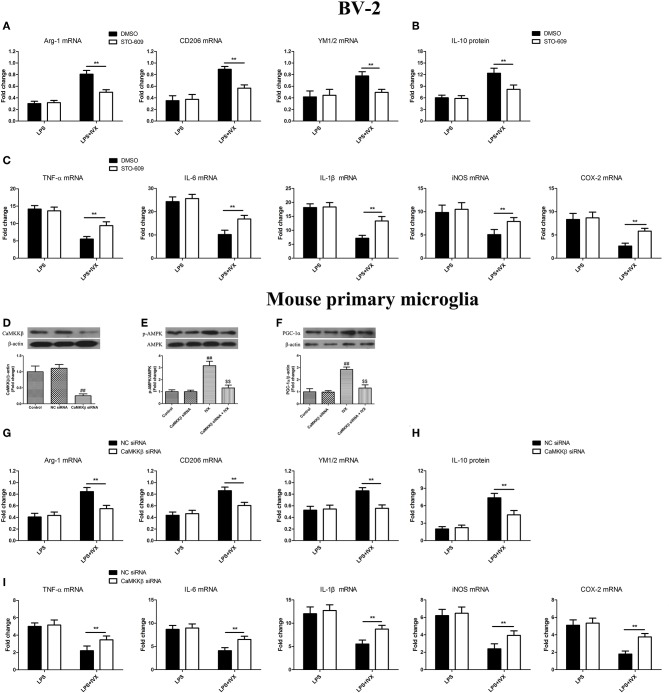
CaMKKβ activation is involved in IVX-mediated microglial polarization in BV-2 cells and mouse primary microglia. **(D)** After treatment with CaMKKβ siRNA for 24 h and then IVX for 2 h in mouse primary microglia, the protein expression of CaMKKβ was measured with Western blotting. **(E)** After treated with CaMKKβ siRNA for 24 h, mouse primary microglia were exposed to IVX for 1 h. The protein expression of p-AMPK was detected by Western blotting. **(F)** After treated with CaMKKβ siRNA for 24 h, mouse primary microglia were exposed to IVX for 3 h. The protein expression of PGC-1α was detected by Western blotting. Following pretreatment with 10 μM STO-609 for 4 h and CaMKKβ siRNA for 24 h, cells were treated with IVX (200 μg/mL) for 2 h and then stimulated with LPS (100 ng/mL) for 6 h. **(B,H)** The mRNA expression of M2 microglial markers (Arg-1, CD206, and YM1/2) were detected via RT-PCR. **(C,I)** The mRNA expression of M1 microglial markers (TNF-α, IL-6, IL-1β, iNOS, and COX-2) were detected via RT-PCR. Pretreatment with 10 μM STO-609 for 4 h and CaMKKβ siRNA for 24 h, cells were treated with IVX (200 μg/mL) for 2 h and then stimulated with LPS (100 ng/mL) for 24 h. **(C,G)** The release of IL-10 was measured with ELISA. The experiments were conducted in triplicate and repeated at least three times. Values are expressed as mean ± SEM (*n* = 4 in each group). ^*##*^*p* < 0.01, vs. control group; ^$$^*p* < 0.01 vs. IVX group; **p < 0.01, STO-609 or CaMKKβ siRNA + LPS + IVX group vs. LPS + IVX group.

CaMKKβ protein expression was decreased in mouse primary microglia when transfected with CaMKKβ siRNA for 24 h (Figure 6D). Pretreatmen with CaMKKβ siRNA attenuated the phosphorylation of AMPK and PGC-1α by IVX in BV-2 cells (Figures 6E,F). In LPS-induced BV-2 cells and mouse primary microglia, 10 μM STO-609 (CaMKKβ inhibitor) and CaMKKβ siRNA did not impact IVX-mediated microglial polarization as measured by the expression of M1 (TNF-α, IL-6, IL-1β, iNOS, and COX-2 mRNA) and M2 (Arg-1, CD206, and YM1/2 mRNA) markers (Figures 6A,C,G,I). In LPS-induced polarized BV-2 cells and mouse primary microglia, pretreatment with STO-609 and CaMKKβ siRNA attenuated the inhibition of M1 markers (TNF-α, IL-6, IL-1β, iNOS, and COX-2 mRNA) by IVX and STO-609 and CaMKKβ siRNA treatment attenuated the enhancement of M2 markers (Arg-1, CD206, and YM1/2 mRNA) by IVX (Figures 6A,C,G,I), suggesting that IVX-mediated CaMKKβ phosphorylation can regulate microglial polarization. In LPS-induced polarized BV-2 cells and mouse primary microglia, pretreatment with STO-609 and CaMKKβ siRNA attenuated the release of IL-10 by IVX (Figures 6B,H), suggesting that IVX enhances M2 microglial polarization via the activation of CaMKKβ.

### IVX Treatment Significantly Decreased LPS-Induced Sickness Behavior and Body Weight Loss and Enhanced Locomotor Activity, in Mice Not Treated With a CaMKKβ or PGC-1α Inhibitor

**Figure 7:**
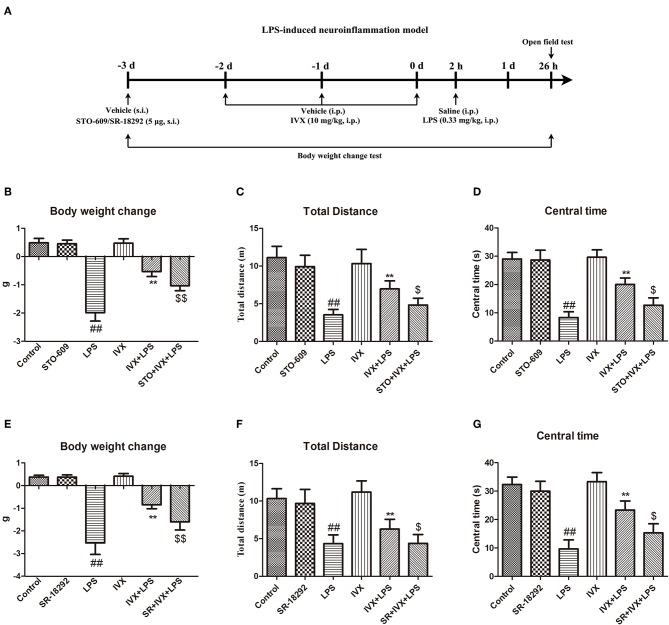
IVX improved LPS-induced sickness behavior in mice. **(A)** Experimental design of a LPS-induced neuroinflammatory model. Mice were injected in the lateral ventricle with 5 μg STO-609 or 5 μg SR-18292 for 24 h, then treated with intraperitoneally injected IVX (10 mg/kg) daily for 3 consecutive days and then subcutaneously injected with LPS (0.33 mg/kg) for 24 h. **(B,E)** The changes in mouse body weight from before to after the experiment. **(C,D,F,G)** The locomotor activity of mice was measured via an open field test. Results are expressed as means ± SEM (*n*= 8–10, in each group). ^*##*^*p* < 0.01, vs. control group; **p< 0.01 vs. LPS group; ^$^*p* < 0.05 and ^$$^*p* < 0.01 vs. IVX + LPS group.

In mice, LPS treatment resulted in body weight loss and pretreatment with IVX inhibited this bodyweight loss (Figures 7B,E); however, intracerebroventricular injection with STO-609 or SR-18292 attenuated the IVX-mediated inhibition of LPS-induced body weight loss (Figures 7B,E). In open field tests, LPS-injected mice experienced locomotor difficulties as indicated by decreased total distance traveled and time spent in the central zone (Figures 7C,D,F,G). IVX treatment improved locomotor activity; however, STO-609 or SR-18292 treatment attenuated this improvement (Figures 7C,D,F,G). IVX-mediated amelioration of LPS-induced sickness behavior in mice was prevented by administration of a CaMKKβ or PGC-1α inhibitor prior to IVX treatment.

### The Anti-inflammatory Effects of IVX on LPS-Injected Mice Is Regulated by the CaMKKβ/AMPK-PGC-1α Signaling Pathway

**Figure 8:**
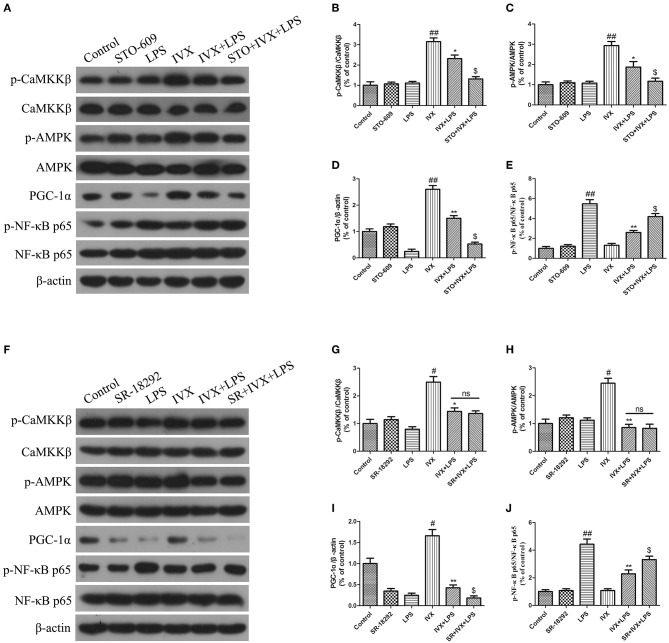
IVX activated the CaMKKβ/AMPK-PGC-1α signaling axis in the cerebral cortices of LPS-injected mice. After the open field test, the cerebral cortices of the mice were harvested. **(A–J)** The protein expression of p-CaMKKβ, p-AMPK, CaMKKβ, AMPK, PGC-1α, NF-κB p65, and p-NF-κB p65 in the cerebral cortices of LPS-injected mice were determined by western blotting. The experiments were conducted in triplicate and repeated at least three times. Values are expressed as mean ± SEM (*n* = 4–5 in each group). ^#^*p* < 0.05 and ^*##*^*p* < 0.01, vs. control group; *p < 0.05 and **p < 0.01 vs. IVX group; ^$^*p*< 0.05 vs. IVX + LPS group.

The effective mechanism of IVX was demonstrated through an IVX-mediated increase in CaMKKβ and AMPK phosphorylation levels, and PGC-1α expression in LPS-injected mice (Figures 8A–D). In LPS-injected mice, STO-609 pretreatment decreased the IVX-mediated CaMKKβ and AMPK phosphorylation levels, and PGC-1α expression (Figures 8A–D). In mice, LPS treatment enhanced phosphorylation of NF-κB p65 and STO-609 pretreatment attenuated the inhibition of NF-κB p65 phosphorylation levels by IVX (Figure 8E). IVX treatment increased CaMKKβ and AMPK phosphorylation levels, and PGC-1α expression in LPS-injected mice (Figures 8A–D,F–I). SR-18292 treatment did not affect the LPS-induced of CaMKKβ and AMPK phosphorylation levels (Figures 8F–I). SR-18292 treatment decreased the IVX-mediated increase in PGC-1α protein expression in LPS-injected mice (Figure 8I). SR-18292 pretreatment attenuated the inhibition of NF-κB p65 phosphorylation levels by IVX in LPS-injected mice (Figure 8J). IVX was anti-inflammatory effects in the presence of LPS-induced neuroinflammation via the regulation of the CaMKKβ/AMPK-PGC-1α/NF-κB signaling pathway.

### IVX Treatment Promotes Microglial Polarization Into M2 Phenotype in LPS-Injected Mouse via Activation of CaMKKβ/AMPK-PGC-1α Signaling Pathway

**Figure 9:**
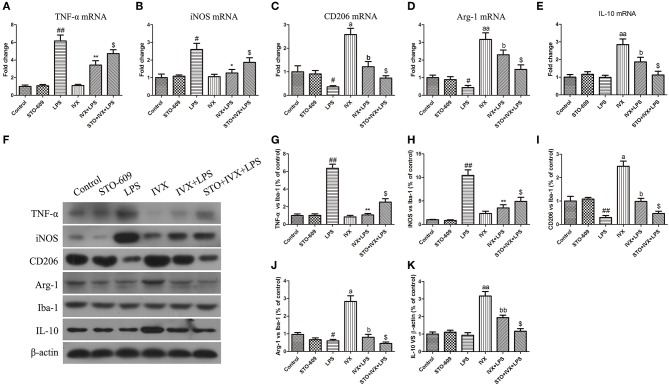
IVX-mediated CaMKKβ activation inhibited M1 microglial polarization and promoted M2 microglial polarization in the cerebral cortices of LPS-injected mice. After the open field test, the cerebral cortices of mice were harvested. To investigate the effect of IVX-mediated CaMKKβ activation on microglial polarization in LPS-injected mice, the mRNA and protein expression of M1 microglial markers (TNF-α and iNOS) and M2 microglial markers (Arg-1 and CD206) were detected via RT-PCR and western blotting. **(A,B)** The mRNA expression of M1 microglial markers (TNF-α and iNOS) were measured via RT-PCR. **(C,D)** The mRNA expression of M2 microglial markers (Arg-1 and CD206) was measured via RT-PCR. **(F–H)** The protein expression of M1 microglial markers (TNF-α and iNOS) were measured via western blotting. **(I,J)** The protein expression of M2 microglial markers (Arg-1 and CD206) was measured via western blotting. **(E,K)** The gene and protein expression of IL-10 was measured via RT-PCR and western blotting. The experiments were conducted in triplicate and repeated at least three times. Values are expressed as mean ± SEM (*n* = 4–5 in each group). ^#^*p* < 0.05 and ^*##*^*p* < 0.01, vs. control group; *p < 0.05 and **p < 0.01 vs. LPS group; ^$^*p*< 0.05 vs. IVX + LPS group; ^*a*^*p* < 0.05 and ^*aa*^*p* < 0.01, vs. control group; ^*b*^*p* < 0.05, ^*bb*^*p* < 0.01 vs. IVX group.

**Figure 10:**
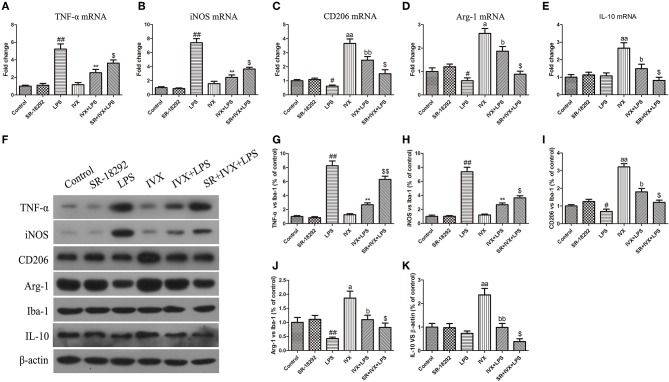
IVX-mediated PGC-1α activation inhibited M1 microglial polarization and promoted M2 microglial polarization in the cerebral cortices of LPS-injected mice. After the open field test, the cerebral cortices of mice were harvested. In LPS-injected mice, to investigate the effect of IVX-mediated PGC-1α activation on microglial polarization, the mRNA and protein expression of M1 microglial markers (TNF-α and iNOS) and M2 microglial markers (Arg-1 and CD206) were tested via RT-PCR and western blotting. **(A,B)** The mRNA expression of M1 microglial markers (TNF-α and iNOS) were measured via RT-PCR. **(C,D)** The mRNA expression of M2 microglial markers (Arg-1 and CD206) was measured via RT-PCR. **(F–H)** The protein expression of M1 microglial markers (TNF-α and iNOS) were measured via western blotting. **(I,J)** The protein expression of M2 microglial markers (Arg-1 and CD206) was measured via western blotting. **(E,K)** The gene and protein expression of IL-10 was measured via RT-PCR and western blotting. The experiments were conducted in triplicate and repeated at least three times. Values are expressed as mean ± SEM (*n* = 4–5 in each group). ^#^*p* < 0.05 and ^*##*^*p* < 0.01, vs. control group; **p< 0.01 vs. LPS group; ^$^*p* < 0.05 and ^$$^*p* < 0.01 vs. IVX + LPS group; ^*a*^*p* < 0.05 and ^*aa*^*p* < 0.01, vs. control group; ^*b*^*p* < 0.05 and ^*bb*^*p* < 0.05 vs. IVX group.

LPS treatment increased the gene and protein expression of M1 markers (TNF-α and iNOS) and reduced the gene and protein expression of M2 markers (Arg-1 and CD206) in LPS-injected mouse (Figures 9, 10). IVX pretreatment suppressed M1 microglia while promoting polarization of microglia to the M2 phenotype in the cerebral cortex. IVX treatment enhanced the expression of M2 markers (Arg-1 and CD206) and STO-609 and SR-18292 pretreatment attenuated the inhibition of M1 markers (TNF-α and iNOS) and enhancement of M2 markers (Arg-1 and CD206) by IVX in LPS-injected mice (Figures 9, 10). Pretreatment with STO-609 and SR-18292 attenuated the inhibition of IVX on IL-10 release in LPS-injected mice (Figures 9E,K, 10E,K). IVX treatment inhibited M1 microglia and enhanced the promotion of microglia to the M2 phenotype in LPS-injected mice by activation of the CaMKKβ/AMPK-PGC-1α signaling pathway.

## Discussion

Our aim was to determine whether IVX suppressed microglia-mediated neuroinflammation by regulating microglial polarization. We found that IVX suppressed pro-inflammatory M1 microglia and promoted anti-inflammatory M2 microglia through polarization via activation of the CaMKKβ/AMPK-PGC-1α signaling axis. Our results indicated that IVX regulated the expression of PGC-1α in BV2 cells via CaMKKβ-dependent AMPK activation (Figure 5) and suggested that IVX improved LPS-induced sickness behavior via CaMKKβ activation and PGC-1α activation (Figure 7).

We found that LPS treatment promoted gene expression of M1 microglia and inhibited gene expression of M2 microglia and caused serious sickness behavior in mice. IVX suppressed M1 microglial polarization and promoted M2 microglial polarization in LPS-activated BV-2 cells and mouse primary microglia and this regulation improved sickness behavior in LPS-treated mice *in vivo*. Our *in vivo* results are supported by our *in vitro* results that revealed IVX promotes microglial polarization into M2 phenotype to exert neuroprotective effect in LPS-caused neuroinflammation.

Fifteen unreported compounds isolated from the rhizomes of Anemarrhena asphodeloides have anti-inflammatory activities (47). It has been reported and summarized that vitexin and isovitexin could be potential substitute medicines for diversity diseases, and may be adjuvants for stubborn diseases or health products (48, 49). However, the effect of isovitexin on brain disorder associated with neuroinflammation hasn't been reported yet. PGC-1α is a transcriptional coactivator that modulates the transcription of many genes associated with cellular metabolism, including mitochondrial biogenesis and respiration, and ROS metabolism (40). IVX enhanced the mRNA and protein expression of PGC-1α and PPARγ in BV-2 cells. PGC-1α activation may inhibit M1 microglial polarization by modulating the NF-κB signaling pathway and promoting M2 microglial polarization through STAT6 and STAT3 signaling pathway activation (21). The neuroprotective effect of IVX was investigated by injecting a PGC-1α inhibitor (SR-18292) into BV-2 cells and the lateral ventricles of mice. We found IVX regulated M1 and M2 microglial polarization to exert a neuroprotective effect through enhancing PGC-1α expression.

AMPK, a major regulator of cellular energy homeostasis, is expressed in a variety of brain cell types, including neurons, microglia and astrocytes and promotes the expression of the M2 microglial phenotype (5, 8–11, 34). We found that IVX increased the phosphorylation of AMPKα in BV-2 cells and the cerebral cortices of mice. siRNA-mediated AMPKα silencing increased mRNA expression of M1 markers (TNF-α and IL-1β) and decreased the mRNA expression of M2 markers (YM1/2) in LPS-activated BV-2 cells (20). The AMPK inhibitor, Compound C, reportedly enhanced M1 microglial polarization and attenuated M2 microglial polarization in the cerebral cortices of mice injected with LPS, however, we found IVX-mediated increases in AMPK phosphorylation in the cerebral cortices of LPS-treated mice (43). This suggests that other signaling pathways indued by IVX are involved in microglial polarization in LPS-injected mice.

AMPK activation is modulated by two upstream kinases, liver kinase B1 (LKB1) and CaMKKβ (50). CaMKKβ is activated by elevated levels of intracellular Ca^2+^ and regulates microglia-mediated neuroinflammation (38). IVX enhanced phosphorylation of CaMKKβ in BV-2 cells and in the cerebral cortices of LPS-injected mice and pretreatment with a CaMKKβ inhibitor (STO-609) attenuated IVX-mediated regulation of microglial polarization. STO-609 pretreatment also attenuated IVX-mediated AMPK and PGC-1α activation; however, CaMKKβ activation was previously reported to produce pro-inflammatory effects in macrophages (51). The effect of CaMKKβ activation on macrophage- and microglia-mediated inflammation requires further study.

## Conclusions

**Figure 11:**
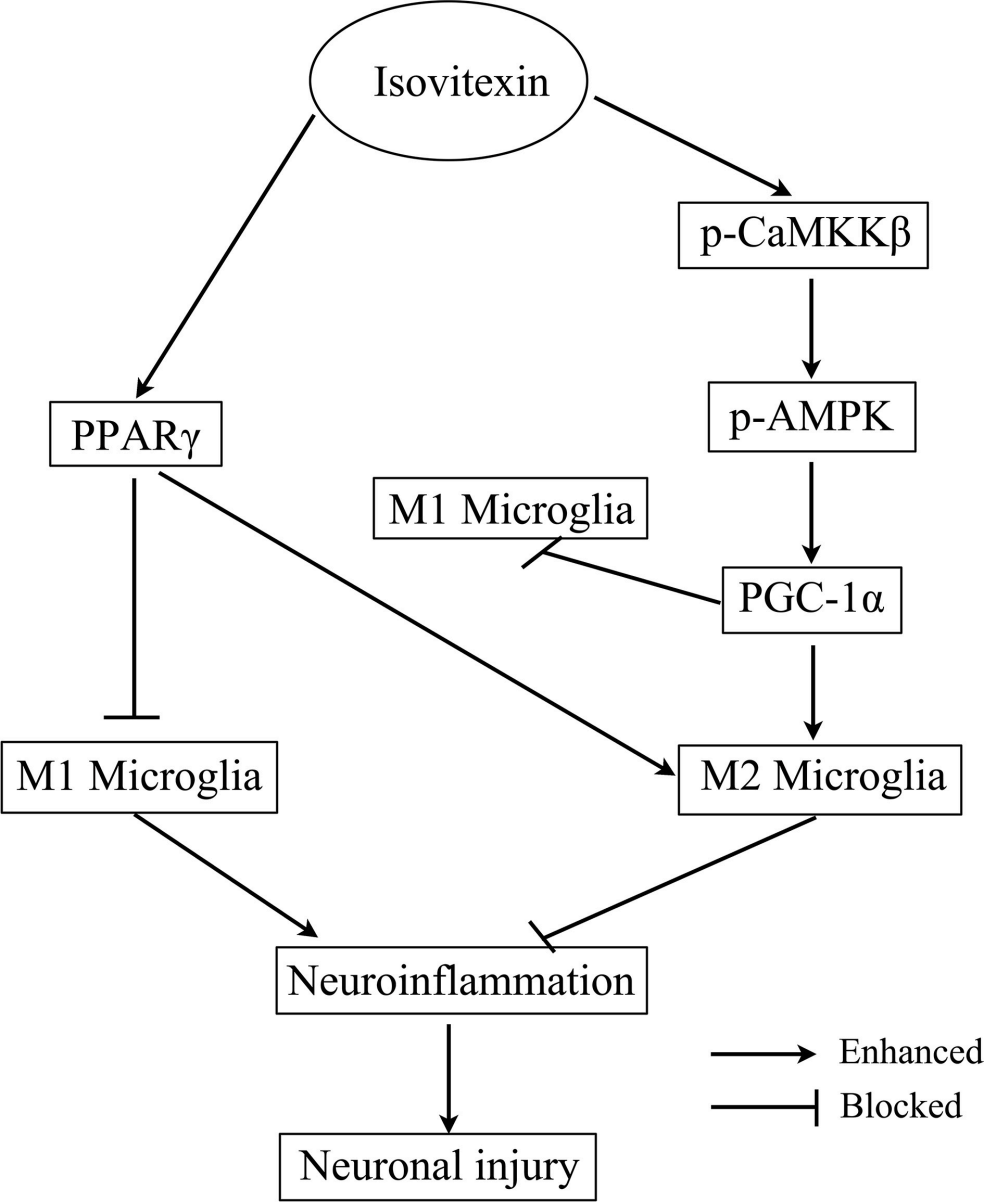
Scheme for the proposed mechanisms of the anti-neuroinflammatory effects of Isovitexin. The mechanisms involve Isovitexin promoting microglia M2 polarization and inhibition of M1 polarization, and the activation of PPARγ and CaMKKβ/AMPK- PGC-1α signaling pathway.

To the best of our knowledge, this is the first study to report that IVX significantly regulated microglial polarization by inhibition of microglia-mediated neuroinflammation via activation of the CaMKKβ/AMPK-PGC-1α signaling axis (Figure 11). Analyzed collectively, our data indicate that IVX could be developed as a therapeutic agent for sickness behavior associated with neuroinflammation. Owing to IVX's multitude of biological functions and its availability as a food source, future research will explore whether IVX can be developed as food supplement to prevent and treat sickness behavior associated with neuroinflammation.

## Data Availability Statement

All datasets generated for this study are included in the article/supplementary material.

## Ethics Statement

This research was conducted according to approved animal protocols and guidelines established by the Institutional Animal Care and Use Committee of Jilin University (Changchun, China) (approved on February 27, 2015; Protocol No. 2015047). We done our best to minimize animal suffering and to reduce the number of animals used.

## Author Contributions

SF and DL designed the research framework. BL, BH, and GH conducted the experiments and wrote this manuscript. DH and XR analyzed the data. YL and JD collected the samples and related information. All authors participated in the manuscript's review and approve the publication of this manuscript.

### Conflict of Interest

The authors declare that the research was conducted in the absence of any commercial or financial relationships that could be construed as a potential conflict of interest.

## Abbreviations

IVX: Isovitexin
LPS: lipopolysaccharide
PPARγ: peroxisome proliferator-activated receptor gamma
PGC-1α: PPARγ coactivator-1α
CNS: central nervous system
Arg1: arginase-1
Ym1: Chitinase-3-like-3
PD: Parkinson's disease
TNF-αm: tumor necrosis factor alpha
IL-1β: interleukin 1β
IL-6: interleukin 6
AMPK: AMP-activated protein kinase
CaMKKβ: calcium/calmodulin-dependent protein kinase kinase beta
CC: Compound C
PS: penicillin-streptomycin
CLB: Cell lysis buffer
DMEM: Dulbecco's Modified Eagle's Medium
FBS: fetal bovine serum
qPCR: Quantitative PCR
AP: anteroposterior
LAT: lateral
DV: dorsoventral
ip.: intraperitoneal
ACSF: artificial cerebrospinal fluid
PVDF: polyvinylidene difluoride
LSD: least significant difference
LKB1: liver kinase B1.
