# Corrigendum: Isovitexin-Mediated Regulation of Microglial Polarization in Lipopolysaccharide-Induced Neuroinflammation via Activation of the CaMKKβ/AMPK-PGC-1α Signaling Axis

**DOI:** 10.3389/fimmu.2020.00041

**Published:** 2020-01-31

**Authors:** Bingrun Liu, Bingxu Huang, Guiqiu Hu, Dewei He, Yuhang Li, Xin Ran, Jian Du, Shoupeng Fu, Dianfeng Liu

**Affiliations:** ^1^College of Animal Science and Veterinary Medicine, Jilin University, Changchun, China; ^2^Laboratory of Biomolecular Research, Division of Biology and Chemistry, Paul Scherrer Institute, Villigen, Switzerland

**Keywords:** isovitexin, microglia polarization, neuroinflammation, microglial markers, M1/M2 microglia

There is an error in the Funding statement. The correct number for National Nature Science Foundation of China is 31772547, 31702211.

The authors apologize for this error and state that this does not change the scientific conclusions of the article in any way. The original article has been updated.

